# Supplementary material for: The Hourglass and the Early Conservation Models—Co-Existing Patterns of Developmental Constraints in Vertebrates
Source: PLoS Genet. 2013 Apr 25;9(4):e1003476. doi: 10.1371/journal.pgen.1003476 (PMC3636041; doi:10.1371/journal.pgen.1003476)
Supplement: Table S1 — P-values from HCNE enrichment analyses. (PDF) [file pgen.1003476.s013.pdf]

Table S1. P-values from HCNE enrichment analyses.

	200bp	**500bp**	1000bp	intron	500bp(90%)
segmentation module	4.0e-3	**8.0e-6**	2.2e-7	2.5e-5	7.9e-1
pharyngula module	1.4e-3	**1.1e-4**	2.3e-7	2.0e-4	6.5e-4

The column in bold corresponds to the case reported in the main text.
