# Supplementary material for: The Hourglass and the Early Conservation Models—Co-Existing Patterns of Developmental Constraints in Vertebrates
Source: PLoS Genet. 2013 Apr 25;9(4):e1003476. doi: 10.1371/journal.pgen.1003476 (PMC3636041; doi:10.1371/journal.pgen.1003476)
Supplement: Table S2 — The list of modules and their enriched GO categories (biological process). (PDF) [file pgen.1003476.s014.pdf]

Table S2. The list of modules and their enriched GO categories (biological process).

GO ID	Term	Annot.	Sign.	Expect.	elim p	bonf p
**Module 1**						
GO:0006468	protein amino acid phosphorylation	446	29	13.7	1.00E-04	7.00E-04
GO:0090244	Wnt receptor signaling pathway involved in somitogenesis	2	2	0.06	9.40E-04	6.58E-03
GO:0006470	protein amino acid dephosphorylation	98	9	3.01	3.09E-03	2.16E-02
GO:0031290	retinal ganglion cell axon guidance	24	4	0.74	5.70E-03	3.99E-02
GO:0043149	stress fiber assembly	5	2	0.15	8.84E-03	6.19E-02
GO:0090090	negative regulation of canonical Wnt receptor signaling pathway	5	2	0.15	8.84E-03	6.19E-02
GO:0021915	neural tube development	31	4	0.95	1.43E-02	9.98E-02
GO:0042451	purine nucleoside biosynthetic process	8	2	0.25	2.33E-02	1.63E-01
GO:0042455	ribonucleoside biosynthetic process	8	2	0.25	2.33E-02	1.63E-01
GO:0046129	purine ribonucleoside biosynthetic process	8	2	0.25	2.33E-02	1.63E-01
**Module 2**						
GO:0016055	Wnt receptor signaling pathway	80	14	4.43	1.10E-04	7.70E-04
GO:0000184	nuclear-transcribed mRNA catabolic process, nonsense-mediated decay	6	4	0.33	1.30E-04	9.10E-04
GO:0042664	negative regulation of endodermal cell fate specification	6	4	0.33	1.30E-04	9.10E-04
GO:0035468	positive regulation of signaling pathway	30	8	1.66	1.70E-04	1.19E-03
GO:0010159	specification of organ position	3	3	0.17	1.70E-04	1.19E-03
GO:0035050	embryonic heart tube development	70	21	3.88	2.00E-04	1.40E-03
GO:0001706	endoderm formation	18	9	1	2.80E-04	1.96E-03
GO:0060218	hemopoietic stem cell differentiation	7	4	0.39	2.90E-04	2.03E-03
GO:0007420	brain development	149	21	8.26	4.10E-04	2.87E-03
GO:0030903	notochord development	31	10	1.72	5.00E-04	3.50E-03
GO:0014028	notochord formation	4	3	0.22	6.50E-04	4.55E-03
GO:0001522	pseudouridine synthesis	9	4	0.5	9.40E-04	6.58E-03
GO:0045893	positive regulation of transcription, DNA-dependent	47	9	2.6	9.50E-04	6.65E-03
**Module 3**						
GO:0009952	anterior/posterior pattern formation	91	19	3.45	1.10E-04	7.70E-04
GO:0048741	skeletal muscle fiber development	14	5	0.53	1.10E-04	7.70E-04
GO:0030510	regulation of BMP signaling pathway	15	5	0.57	1.70E-04	1.19E-03
GO:0043049	otic placode formation	15	5	0.57	1.70E-04	1.19E-03
GO:0030901	midbrain development	15	5	0.57	1.70E-04	1.19E-03
GO:0021523	somatic motor neuron differentiation	4	3	0.15	2.10E-04	1.47E-03
GO:0042694	muscle cell fate specification	4	3	0.15	2.10E-04	1.47E-03
GO:0021508	floor plate formation	9	4	0.34	2.20E-04	1.54E-03
GO:0033334	fin morphogenesis	57	9	2.16	2.60E-04	1.82E-03
GO:0007156	homophilic cell adhesion	58	9	2.2	3.00E-04	2.10E-03
GO:0007517	muscle organ development	59	16	2.23	3.00E-04	2.10E-03
GO:0007169	transmembrane receptor protein tyrosine kinase signaling pathway	71	10	2.69	3.10E-04	2.17E-03
GO:0009888	tissue development	329	41	12.46	3.40E-04	2.38E-03
GO:0031016	pancreas development	39	7	1.48	5.70E-04	3.99E-03
GO:0030182	neuron differentiation	156	27	5.91	5.90E-04	4.13E-03
GO:0009953	dorsal/ventral pattern formation	65	9	2.46	7.10E-04	4.97E-03
GO:0007399	nervous system development	326	60	12.34	8.40E-04	5.88E-03

Continued on next page

Table S2 – continued from previous page

GO ID	Term	Annot.	Sign.	Expect.	elim p	bonf p
GO:0007223	Wnt receptor signaling pathway, calcium modulating pathway	21	5	0.8	9.30E-04	6.51E-03
GO:0021984	adenohypophysis development	9	5	0.34	9.70E-04	6.79E-03
GO:0001708	cell fate specification	36	9	1.36	1.06E-03	7.42E-03
**Module 4**						
GO:0030154	cell differentiation	422	39	13.18	1.20E-04	8.40E-04
GO:0030902	hindbrain development	57	11	1.78	2.30E-04	1.61E-03
GO:0050769	positive regulation of neurogenesis	12	4	0.37	3.80E-04	2.66E-03
GO:0048663	neuron fate commitment	13	4	0.41	5.30E-04	3.71E-03
GO:0048593	camera-type eye morphogenesis	47	9	1.47	8.60E-04	6.02E-03
GO:0030900	forebrain development	51	7	1.59	9.60E-04	6.72E-03
GO:0051091	positive regulation of transcription factor activity	7	3	0.22	9.60E-04	6.72E-03
GO:0030901	midbrain development	15	4	0.47	9.70E-04	6.79E-03
GO:0021915	neural tube development	31	5	0.97	2.50E-03	1.75E-02
GO:0002043	blood vessel endothelial cell proliferation involved in sprouting angiogenesis	3	2	0.09	2.86E-03	2.00E-02
**Module 5**						
GO:0007602	phototransduction	10	4	0.28	1.10E-04	7.70E-04
GO:0006813	potassium ion transport	79	9	2.18	3.10E-04	2.17E-03
GO:0018298	protein-chromophore linkage	13	4	0.36	3.40E-04	2.38E-03
GO:0007156	homophilic cell adhesion	58	7	1.6	1.03E-03	7.21E-03
GO:0006836	neurotransmitter transport	36	8	1	1.62E-03	1.13E-02
GO:0006814	sodium ion transport	52	6	1.44	2.96E-03	2.07E-02
GO:0007267	cell-cell signaling	41	8	1.13	3.14E-03	2.20E-02
GO:0007194	negative regulation of adenylate cyclase activity	5	2	0.14	7.21E-03	5.05E-02
GO:0007268	synaptic transmission	21	6	0.58	1.04E-02	7.25E-02
GO:0006208	pyrimidine base catabolic process	6	2	0.17	1.06E-02	7.43E-02
**Module 6**						
GO:0006805	xenobiotic metabolic process	3	2	0.05	9.20E-04	6.44E-03
GO:0006584	catecholamine metabolic process	6	2	0.11	4.44E-03	3.11E-02
GO:0019882	antigen processing and presentation	20	3	0.35	4.95E-03	3.47E-02
GO:0006022	aminoglycan metabolic process	22	3	0.39	6.52E-03	4.56E-02
GO:0046686	response to cadmium ion	8	2	0.14	8.10E-03	5.67E-02
GO:0009607	response to biotic stimulus	47	4	0.83	9.29E-03	6.50E-02
GO:0000272	polysaccharide catabolic process	9	2	0.16	1.03E-02	7.21E-02
GO:0006026	aminoglycan catabolic process	9	2	0.16	1.03E-02	7.21E-02
GO:0055114	oxidation reduction	409	14	7.23	1.35E-02	9.42E-02
GO:0006144	purine base metabolic process	11	2	0.19	1.54E-02	1.08E-01
**Module 7**						
GO:0043687	post-translational protein modification	748	16	8.26	7.70E-03	5.39E-02
GO:0050896	response to stimulus	622	16	6.87	9.40E-03	6.58E-02
GO:0051707	response to other organism	40	3	0.44	9.60E-03	6.72E-02
GO:0006950	response to stress	329	9	3.63	1.04E-02	7.28E-02
GO:0006508	proteolysis	391	10	4.32	1.10E-02	7.70E-02
GO:0051715	cytolysis of cells of another organism	1	1	0.01	1.10E-02	7.70E-02
GO:0044403	symbiosis, encompassing mutualism through parasitism	1	1	0.01	1.10E-02	7.70E-02

Continued on next page

Table S2 – continued from previous page

GO ID	Term	Annot.	Sign.	Expect.	elim p	bonf p
GO:0051801	cytolysis of cells in other organism involved in symbiotic interaction	1	1	0.01	1.10E-02	7.70E-02
GO:0031640	killing of cells of another organism	1	1	0.01	1.10E-02	7.70E-02
GO:0070193	synaptonemal complex organization	1	1	0.01	1.10E-02	7.70E-02

Annot. — total number of genes annotated with a given GO category; Sign. — number of (significant) genes in the module annotated with a given GO category; Expect. — expected number of genes in the module annotated with a given GO category; elim p — P-value from “elim” algorithm of topGO, bonf p — P-value after Bonferroni correction.
