# Supplementary material for: The Hourglass and the Early Conservation Models—Co-Existing Patterns of Developmental Constraints in Vertebrates
Source: PLoS Genet. 2013 Apr 25;9(4):e1003476. doi: 10.1371/journal.pgen.1003476 (PMC3636041; doi:10.1371/journal.pgen.1003476)
Supplement: Text S1 — Supplementary analyses. (PDF) [file pgen.1003476.s016.pdf]

Supplementary Materials: The hourglass and the early conservation models — co-existing patterns of developmental constraints in vertebrates

Barbara Piasecka[1,2,4], Paweł Lichocki[3], Sébastien Moretti[1,5], Sven Bergmann[2,4,#] Marc Robinson-Rechavi[1,4,#,*]
1 Department of Ecology and Evolution, University of Lausanne, Lausanne, Switzerland
2 Department of Medical Genetics, University of Lausanne, Lausanne, Switzerland
3 Laboratory of Intelligent Systems, EPFL, Lausanne, Switzerland
4 Swiss Institute of Bioinformatics, Lausanne, Switzerland
5 Vital-IT, Swiss Institute of Bioinformatics, Lausanne, Switzerland
# These authors contributed equally to this work.
∗ E-mail: marc.robinson-rechavi@unil.ch

Re-analysis of previous studies

Domazet-Lošo and Tautz (2010)

In a recent paper, Domazet-Lošo and Tautz [1] suggested that *'the phylotypic stage does express the oldest transcriptome set and that younger sets are expressed during early and late development"*. To study the relationship between gene expression, ontogeny and phylogeny, the authors proposed a measure called the "transcriptome age index" (TAI). In the main text (Box 1) we show that the transcriptome age measured with TAI [1] differs strongly if the log10-transformation of the data is applied. Here, we first discuss the advantages of log-transformation, and next we show that also applying several other measures and transformations of the data never reproduces the results reported in [1]. On the contrary, we find always that the age of the transcriptome decreases during development.

The microarray signal intensity values that were used in [1] display a log-normal distribution and span from 1 to 10^5 (figure S1). If one uses non-transformed data to calculate TAI, then the five orders of magnitude of difference between expressions of highly and lowly expressed genes translates into five orders of magnitude of difference of the weights of the phylogenetic ranks. In practice, this means that highly expressed genes are given a very high importance, whereas lowly expressed genes are given almost none (figure S2). It is disputable whether this is a correct interpretation of the biological reality, because even lowly expressed genes (which are a large majority) do play a role in the development and are shaped by evolutionary forces. Thus, one should not neglect them, if one wishes to interpret the TAI profile in the context of evolutionary constraints or evolutionary adaptation on the whole transcriptome, as in [1]. It can also be legitimate to study only a subset of genes, but then this should be done explicitly, and the properties of this subset should be well defined. In order to take into account all genes having a function during development, the data must be transformed, so that the weights of the phylogenetic ranks span a more comparable range. Of note, X-fold difference in signal intensity does not necessarily imply X-fold difference in RNA concentration [2].

Moreover, non-log-transformed data are very sensitive to outliers. We identified the probe A_15_P161596 as an outlier (figure S3A) which strongly distorts the TAI profile reported in [1]. If this single outlier is removed, a TAI peak during gastrulation – which in [1] was given an evolutionary interpretation and linked to the action of the group of genes that emerged in Metazoa – disappears and leaves the gastrulation trend less marked (figure S3B). In contrast, the presence of the outlier has little, if any, influence on the TAI profile calculated on log-transformed data (figure S3C), showing how the log-transformation leads to a more robust analysis.

Also, in [1], the authors used all 16 188 probes to calculate TAI. Since some of them map to the same gene, this results in signal multiplication for some phylostrata. To overcome this problem, we calculated TAI on data with averaged signal from probes mapped to the same gene. This changes the TAI pattern

observed by the authors [1]: the oldest transcriptome now seem to be expressed in mid-larval stage, instead of the phylotypic stage (figure S4A). In contrast, the TAI profile calculated on log-transformed data is more robust, as the pattern remains unchanged and does not depend on mapping to probes or genes (figure S4B).

Another approach to reduce the effect of highly expressed genes is to treat all expressed genes as equally important, i.e., recode as present-absent. This recovers the same pattern as log-transformation (figure S5). Of note, this approach was suggested in [1], without discussion of the results.

Finally, we searched for alternative measures of the evolutionary age of the transcriptome over ontogeny. We computed: (i) the difference in median expression profile of old genes vs. young genes (figure S6A) (similar to [3]); and (ii) the mean age of expressed genes (figure S6B). Both measures recover the decreasing trend over ontogeny. Moreover, measure (i) confirms that the male transcriptome is younger than the female one, consistent with the known fast evolution of male-specific genes [4], whereas the original analysis [1] indicated the opposite - younger female transcriptome.

Overall, it seems that the transcriptomic hourglass pattern reported previously [1] is not robust to different methods of analysis.

Quint et al. (2012)

The methodology developed in [1] was recently used in a study by Quint et al. [5]. The authors used TAI to measure the transcriptome age over the development of *Arabidopsis thaliana*, and an analogous measure, transcriptome divergence index (TDI), to measure transcriptome sequence conservation over development. They report that genes expressed at the torpedo stage are older and more conserved in sequence. The study suffers from the same methodological issues as discussed in the previous section. The weights used to compute TAI and TDI were raw signal intensities measured on the microarrays, which differed by more than two orders of magnitude between lowly and highly expressed genes. Even though this difference was lower than in the study of Domazet-Lošo and Tautz [1], the effect of the most expressed genes on the final pattern was even more remarkable. We found that excluding only 1% of top expressed genes in each developmental stage of *Arabidopsis* changes both TAI and TDI patterns such that they no longer support the hourglass hypothesis (figure S7). Similarly, both TAI and TDI patterns no longer support the hourglass (nor indeed any pattern) if they are calculated on log-transformed intensity values (figure S8).

Irie and Kuratani (2011)

Another analysis suggested that expression diverges less between vertebrate species in the phylotypic stage [6]. The authors calculated Spearman correlations between expression profiles of genes of four species: mouse, zebrafish, chicken and frog. They calculated these correlations for all possible pairs of stages, because it was not obvious how to map developmental stages between species. The correlations between expression profiles of genes were reported to be strongest on average at mid-development, supporting the hourglass model.

Here, we reproduced these results for three species: mouse, zebrafish and chicken. We did not reanalyze the frog data, because the expression was measured for tetraploid *Xenophus laevis*, whereas genome annotation available in Ensembl comes from diploid *Xenophus tropicalis*.

We first divided the development of three species into three general stages: early, middle and late (figure S9). The middle stage contained the time points from the phylotypic stage. The early stage contained the time points preceding the phylotypic stage. And, the late stage contained the time points following the phylotypic stage. We excluded from the analysis the first time point of mouse and zebrafish development, as they had no corresponding time point in the chicken development.

We verified if the middle stage displayed a higher expression similarity than the early stage. To this aim, for each pair of species, we compared the Spearman correlation values between all time points

from the early stages of the two species with the Spearman correlation values between all time points from the middle stages of the two species (field A vs. field B on figure S9). We detected a statistically significant difference only for mouse and chicken (Mann-Whitney U test, $p = 0.018$). However, because the time points from mid and late mouse stages displayed high correlation with almost any chicken time point, we performed a randomization test to confirm the significance of our observation. We permuted the order of chicken time points and compared again the correlation values between early and middle stages. Notably, among the 100 randomizations as many as 43 comparisons had P-value lower than the previously observed $p = 0.018$. Overall, the pattern of presumably conserved gene expression in middle development, reported in [6], was not significant for any pair of species.

Artificial expression profiles for the ISA

We initialized the ISA with seven artificial expression profiles corresponding to consecutive developmental stages. Our main goal was to compare genes expressed in early, mid and late development. The early genes are known to divide into maternal genes (pre-MBT) and zygotic genes (post-MBT) [7]. Consequently, we originally envisioned four artificial expression profiles: pre-MBT, post-MBT, middle and late. During the ISA run, these profiles resulted in four modules containing genes with expression limited to cleaveage/blastula, gastrula, segmentation and juvenile stages, respectively. To cover the entire development we added three other artificial profiles corresponding to the missing stages (pharyngula, larva and adult) and we run ISA again. The seven profiles used to run the ISA are shown on the figure S10.

Gene sequence analysis - purifying selection

In addition to the average d_N/d_S per gene, we have also narrowed our analysis only to the sites under strong purifying selection. For every module we calculated the median ω_0 (and the median p_0) of its k genes, where k was the number of genes belonging to one of the 5772 gene trees (see Methods). Next, we generated 10 000 sets of k randomly chosen genes. For each set we calculated the median ω_0 (and the median p_0). Thus, we constructed a sampling distribution of the median ω_0 (and the median p_0) values for a set of k genes. Then we calculated the probability that the median ω_0 (and the median p_0) of the original module was sampled from the constructed distribution. This allowed us to assess if the observed median ω_0 (and the observed median p_0) was significantly different from the expected median value. To correct for multiple testing we applied the Bonferroni correction. We used 0.01 as a significance level.

We found that purifying selection was significantly lower during post-embroynic stages than over embryonic development. This is supported by both a lower percentage of sites under purifying selection (figure S11A) and higher values of ω_0 (figure S11B) for genes expressed in juvenile and adults.

Sequence conservation between mouse and human

The orthology relationships, and the d_N and d_S values were obtained from Ensembl version 63 [8]. We retrieved 6039 zebrafish genes with one-to-one orthologs in mouse and human (the estimated divergence time is 61.5 MYA between the two mammalian species and 416 MYA with *Danio rerio* [9]) and the pairwise d_N/d_S between mouse and human genes using Biomart [10]. For every module we calculated the median d_N/d_S ratio of its k genes, where k was the number of genes having one-to-one relationship with mouse and human genes. Next, we generated 10 000 sets of k randomly chosen genes. For each set we calculated the median d_N/d_S ratio. Thus, we constructed a sampling distribution of the median d_N/d_S values for a set of k genes. Then we calculated the probability that the median d_N/d_S of the original module was sampled from the constructed distribution. It allowed us to assess if the observed

median d_N/d_S ratio was significantly different from the expected median value. To correct for multiple testing we applied the Bonferroni correction. We used 0.01 as a significance level.

We found a good agreement between results reported in the main text and for mouse-human orthologs (figure S12).

Highly conserved non-coding elements

We tested the sensitivity of the observed enrichment of HCNEs for genes expressed in mid-development, reported in the main text. To this aim, for each of the 14 293 Ensembl genes considered in our analysis, we calculated the number of HCNEs (70% identity) in regions of 200, and 1000 base pairs upstream from the transcription start site (TSS), as well as in the intronic regions. Also, we repeated the analysis looking for HCNEs in regions of 500 bp upstream from the transcription start site (as in the main text), but for HCNEs of 90% identity. To this aim we downloaded and used the file *HCNE_danRer7_mm9_90pc_50col.bed.gz*. Other settings and the statistical analysis were the same as in the main text (see Methods). The results of all four additional analyses are in a good agreement with the results reported in the main text (table S1).

Microsynteny conservation

We checked for modules' enrichment in genes belonging to conserved ancestral microsyntenic pairs (CAMPs) [11]. From the list of 260 zebrafish CAMPs (Irimia, private communication) we selected 75 gene pairs involved in developmental regulation, i.e., "bystander gene + trans-dev gene". Both, bystander and trans-dev genes were reported to have conserved introns sequences. Thus, the trans-dev genes could potentially overlap with genes for which we detected enrichment in HCNEs in introns, as well as in the regions 1000 bp upstream from the TSS (CAMPs were shown to have very short intergenic regions, in some cases < 1kb). We crossed the list of trans-dev genes with the list of genes from each module. We performed hypergeometric test to assess if the overlap between genes was significant. To correct for multiple testing we applied the Bonferroni correction. The number of CAMP-trans-dev genes in the seven modules were the following: $1, n.s.; 6, n.s.; 7, p = 0.018; 4, n.s.; 2, n.s.; 1, n.s.; 0, n.s.$ The overrepresentation of trans-dev genes in the segmentation module stays in agreement with enrichment in HCNE detected in introns and in regions 1000 bp upstream from the TSS for genes belonging to this module. We also checked for enrichment in the remaining 185 CAMPs. Although they were reported to often be co-expressed, we did not find any such pair in our modules.

References

1. Domazet-Lošo T, Tautz D (2010) A phylogenetically based transcriptome age index mirrors ontogenetic divergence patterns. Nature 468: 815-8.

2. Kahn K (2008) Tutorial: Introduction to DNA Microarrays. http://www.chem.ucsb.edu/~kalju/chem162/public/genechip_intro.html.

3. Roux J, Robinson-Rechavi M (2008) Developmental constraints on vertebrate genome evolution. PLoS Genet 4: e1000311.

4. Ellegren H, Parsch J (2007) The evolution of sex-biased genes and sex-biased gene expression. Nature Reviews Genetics 8: 689–698.

5. Quint M, Drost HG, Gabel A, Ullrich KK, Bönn M, et al. (2012) A transcriptomic hourglass in plant embryogenesis. Nature 490: 98-101.

6. Irie N, Kuratani S (2011) Comparative transcriptome analysis reveals vertebrate phylotypic period during organogenesis. Nat Commun 2: 248.

7. Aanes H, Winata CL, Lin CH, Chen JP, Srinivasan KG, et al. (2011) Zebrafish mRNA sequencing deciphers novelties in transcriptome dynamics during maternal to zygotic transition. Genome Res 21: 1328-38.

8. Hubbard TJP, Aken BL, Ayling S, Ballester B, Beal K, et al. (2009) Ensembl 2009. Nucleic Acids Res 37: D690-7.

9. Benton MJ, Donoghue PCJ (2007) Paleontological evidence to date the tree of life. Mol Biol Evol 24: 26-53.

10. Smedley D, Haider S, Ballester B, Holland R, London D, et al. (2009) BioMart – biological queries made easy. BMC Genomics 10: 22.

11. Irimia M, Tena JJ, Alexis M, Fernandez-Miñan A, Maeso I, et al. (2012) Extensive conservation of ancient microsynteny across metazoans due to cis-regulatory constraints. Genome Res .

12. LeProust E (2008) Agilent's Microarray Platform: How High-Fidelity DNA Synthesis Maximizes the Dynamic Range of Gene Expression Measurements. Application Note - Agilent Technologies 5989-9159EN. http://www.chem.agilent.com/en-US/Search/Library/_layouts/Agilent/PublicationSummary.aspx?whid=56080&liid=2024.

13. Wolf YI, Novichkov PS, Karev GP, Koonin EV, Lipman DJ (2009) The universal distribution of evolutionary rates of genes and distinct characteristics of eukaryotic genes of different apparent ages. Proc Natl Acad Sci U S A 106: 7273-80.

Figures

Figure S1. Total distribution of signal intensity from all 140 microarrays [1].

Figure S2. TAI hourglass pattern in zebrafish development [1] is driven by the subset of most highly expressed genes. Removing the 20% of top expressed genes at every developmental stage changes the overall pattern. Resulting TAI pattern has very low values and does not follow the hourglass shape any more (grey line).

Figure S3. Sensitivity to outliers. (A) Raw expression signal of probe A_15_P161596 across zebrafish development. (B) TAI calculated on non-transformed data across zebrafish development without this probe (red) and the effect of this probe on TAI pattern (grey). (C) TAI calculated on log10-transformed data across zebrafish development without this probe (red) and the effect of this probe on TAI pattern (grey). Expression data from [1].

Figure S4. TAI calculated using expression intensities of genes, instead of probes, across zebrafish development. For each gene we averaged the signal intensity from all corresponding probes. After this process 16 188 probes' intensities values were reduced to 12 892 genes' intensities values, which were used to weight the phylogenetic ranks of genes (if two different phylostrata were assigned to the same gene, the older one was chosen). (A) non-transformed data was used. (B) log10-transformed data was used. Expression data from [1].

Figure S5. TAI calculated using genes recoded as present-absent across zebrafish development. At a given stage of development, if the log10-intensity value of a gene is above one [12], its expression is set to 1, otherwise it is set to 0. Other notations as in figure 1 (in main text). Expression data from [1].

Figure S6. Alternative measures of transcriptome age. (A) Mean age of genes expressed across zebrafish development; age estimated with the TimeTree database (*www.timetree.org*). A gene is considered expressed at a given stage of development if its log10-intensity is above one [12]. (B) Difference between median expression profiles of old genes and young genes across zebrafish development. Here, the genes that have emerged before the evolution of Metazoa are considered old and the genes that have emerged since the ancestor of Euteleostomi are considered young. The difference between the two groups is always positive, reflecting that old genes tend to be more expressed than young genes [13]. The results are robust to the choice of cutoffs used to define old and young genes (data not shown). Red dashed line - female data, blue dashed line - male data. Other notations as in figure 1 (main text). Expression data from [1].

Figure S7. TAI and TDI hourglass patterns in *Arabidopsis* development [5] are driven by a very small subset of the most highly expressed genes. Removing only the 1% of top expressed genes at each developmental stage changes the overall pattern. Resulting TAI and TDI patterns do not follow the hourglass shape any more (grey line).

Figure S8. TAI and TDI calculated using raw (green line) and log-transformed (grey line) expression signal intensities. Data from [5].

Figure S9. Correlation between expression levels of genes across developmental time points of mouse, chicken and zebrafish. Field A denotes the early stages, field B denotes the phylotypic stages, and field C denotes the late stages of development. Expression data from [6].

Figure S10. Artificial expression profiles used to initialize the ISA: pre-MBT, post-MBT, "middle", pharyngula, larva, "late", adult. These profiles resulted in modules containing genes expressed specifically in: cleavage/blastula, gastrula, segmentation, pharyngula, larva, juvenile, and adult, respectively.

Figure S11. Measures of purifying selection for gene trees of bony fishes. (A) Average dN/dS for sites under purifying selection (ω_0). (B) Proportion of sites under purifying selection (p_0).

Figure S12. d_N/d_S ratio for human-mouse one-to-one orthologs. The orthologs were obtained by projecting the genes expressed in the zebrafish modules to their one-to-one orthologs in mouse and human.

Tables

Table S1. P-values from HCNE enrichment analyses.
Table S2. The list of modules and their enriched GO categories (biological process).
Table S3. The list of genes belonging to each module.
